# Performance of regional oxygen saturation monitoring by near-infrared spectroscopy (NIRS) in pediatric inter-hospital transports with special reference to air ambulance transports: a methodological study

**DOI:** 10.1007/s10877-017-0094-z

**Published:** 2017-12-28

**Authors:** Tova Hannegård Hamrin, Peter J. Radell, Urban Fläring, Jonas Berner, Staffan Eksborg

**Affiliations:** 1 Department of Physiology and Pharmacology, Section of Anesthesiology and Intensive Care, Karolinska Institutet, Astrid Lindgren Children’s Hospital, Karolinska University Hospital Solna, Stockholm, Sweden; 2 Childhood Cancer Research Unit Q6:05, Department of Women’s and Children’s Health, Karolinska Institutet, Astrid Lindgren Children’s Hospital, Karolinska University Hospital Solna, Stockholm, Sweden; 3 Pediatric Perioperative Medicine and Intensive Care, Astrid Lindgren Children’s Hospital, Karolinska University Hospital Solna, 171 76 Stockholm, Sweden

**Keywords:** Near-infrared spectroscopy (NIRS), Monitoring, Savitzky–Golay algorithm, Pediatric, Inter-hospital transports, Air-ambulance

## Abstract

The aim of the present study was to evaluate the performance of regional oxygen saturation (rSO_2_) monitoring with near infrared spectroscopy (NIRS) during pediatric inter-hospital transports and to optimize processing of the electronically stored data. Cerebral (rSO_2_-C) and abdominal (rSO_2_-A) NIRS sensors were used during transport in air ambulance and connecting ground ambulance. Data were electronically stored by the monitor during transport, extracted and analyzed off-line after the transport. After removal of all zero and floor effect values, the Savitzky–Golay algorithm of data smoothing was applied on the NIRS-signal. The second order of smoothing polynomial was used and the optimal number of neighboring points for the smoothing procedure was evaluated. NIRS-data from 38 pediatric patients was examined. Reliability, defined as measurements without values of 0 or 15%, was acceptable during transport (> 90% of all measurements). There were, however, individual patients with < 90% reliable measurements during transport, while no patient was found to have < 90% reliable measurements in hospital. Satisfactory noise reduction of the signal, without distortion of the underlying information, was achieved when 20–50 neighbors (“window-size”) were used. The use of NIRS for measuring rSO_2_ in clinical studies during pediatric transport in ground and air-ambulance is feasible but hampered by unreliable values and signal interference. By applying the Savitzky–Golay algorithm, the signal-to-noise ratio was improved and enabled better post-hoc signal evaluation.

## Introduction

Near-infrared light passes through tissues such as skin and bone with minimal absorption, the two main absorbers of near-infrared light in blood being oxyhemoglobin (HbO_2_) and deoxyhemoglobin (HbR) [1]. The difference in absorption of HbO_2_ and HbR is measured and represents the regional oxygen saturation (rSO_2_) of the tissue. Since most blood in tissue is post-arteriolar the rSO_2_ is an estimate of regional venous saturation. Universal normal rSO_2_ values applicable to all patients have not been established since the proportion of venous/arterial blood in human tissue has an inter-patient variation in combination with limitations in the measuring technique [2].

Near-infrared spectroscopy (NIRS)-monitoring is attractive in neonatal and pediatric practice since the penetration of the signal into the tissue corresponds well with the anatomy of neonates, infants and children [3], i.e. less subcutaneous fat, thinner muscle walls and bones. Pediatric studies have demonstrated good correlation between cerebral rSO_2_ and jugular venous bulb saturation [4]. Abdominal rSO_2_ has shown strong correlation with gastric intra-mucosal pH [5]. Multisite NIRS monitoring has been advocated to provide insight into the tissue response to different types of clinical interventions [6]. NIRS is widely used in different clinical settings including pediatric and adult cardiac surgery and neonatal and pediatric intensive care. Increasing interest has been drawn to the use of NIRS monitoring in the pre-hospital area since it is non-invasive and easy to use [7]. There are few studies of NIRS utilization in a transport environment and only two concerning pediatric patients [8, 9]. These studies suggest that cerebral oxygenation monitoring with NIRS can be used in a transport environment and that NIRS might be a useful complement to existing monitoring during inter-hospital transports [8–11]. In the two studies concerning pediatric patients the same type of monitor was used (INVOS 5100C), but a number of monitors have been used in different studies.

NIRS-values are usually derived from real time readings on-line, but data can be stored by the monitor, for example during transport, and extracted and examined later off-line. Our experience has been that real time readings are sometimes unreliable during transport and that individual values vary greatly, resulting in uncertainty. Noise reduction remains the most important factor in improving accuracy and precision of signal interpretation in vivo [6].

To our knowledge, earlier studies of NIRS utilization in a transport environment have been carried out using monitor readings on line and not by analyzing the electronically stored data.

The aim of the present study was to evaluate the performance of rSO_2_ monitoring with NIRS during pediatric inter-hospital transport and to optimize processing of the electronically stored data.

## Methods

### Study design and sample

This is a methodological study, registered in the Australian New Zealand Clinical Trials Registry (ANZCTR) with registration number ACTRN12617000750381. Following ethical approval and written parental informed consent, 38 critically ill children scheduled for inter-hospital transport by a specialized pediatric transport team at the Pediatric Intensive Care Unit (PICU) at Astrid Lindgren Children’s Hospital, Karolinska University Hospital in Stockholm, were enrolled in the study between January 2014 and September 2016 (convenience sampling). Exclusion criteria were lack of consent or participation in any other clinical research study. Transports were both acute and planned transfers to and from the PICU at Astrid Lindgren Children’s Hospital. The team is staffed by a PICU consultant and a specialist anesthesia or intensive care registered nurse with a minimum of 3 years experience in pediatric anesthesia or pediatric intensive care [12].

### Equipment and procedures

In all patients, a cerebral sensor was placed over the forehead and a somatic sensor was placed in the infra-umbilical area for abdominal regional oxygen saturations, rSO_2_-C and rSO_2_-A respectively (INVOS-5100C, Covidien, Mansfield, MA, USA). The sensors had the following dimensions: 17.25 cm^2^ for neonates and infants and 28.8 cm^2^ for pediatric patients. The probes had two light paths with an emitter/diode spacing of 30–40 mm and a light penetrating depth of 20–40 mm. Monitoring began at the hospital before patient transport and was continued during transfer in ground ambulance to and from the airport as well as during air ambulance transport and was finished upon arrival at the receiving hospital. For some patients, NIRS monitoring was done for a period of hours prior to transport. Cerebral and abdominal rSO_2_ data were stored by the INVOS monitor during transport and extracted and analyzed off-line after the transport. To reduce ambient light exposure, aluminum foil was used to cover the cerebral probe and the abdominal probe was covered under the patient´s clothes and blankets. The external battery time of the INVOS 5100C is approximately 20 min, which made access to an external power supply for both ground and air ambulance necessary.

The NIRS data was downloaded from the monitor using an Excel spreadsheet (Microsoft Excel 2010). For each patient, the number of “zero values” was identified for both the cerebral and abdominal sensors during the entire monitoring sequence. The number of values which remained steady at the lowest noted detection level of 15% were also identified and defined as “floor effect” values.

After removal of all zero and floor-effect values, the Savitzky–Golay algorithm of smoothing and differentiation of data by simplified least square procedures (least-squares fitting) was used on the stored data to perform noise reduction in the signal and thereby enable better signal evaluation [13]. The second order of smoothing polynomial was used and the optimal number of neighboring points (“window-size”) for the smoothing procedure was determined to avoid distortions of the signal such as reduction of amplitude or broadening of narrow peaks in the recorded data. MATLAB®, MathWorks (Natick, MA, USA) was applied for the implementation and analysis of the Savitzky–Golay filters. The data points had a spacing of 6 s.

To find an optimal value for the numbers of neighbors, we investigated the variability in the signal by using the absolute difference between two adjacent readings. An overview was achieved by using 50–100 neighbors. To obtain more detailed information, the number of neighbors was reduced when the time frame was shortened.

### Statistical analysis

Data is presented as median and inter-quartile range. The NIRS curves were smoothed by the Savitzky–Golay filtering method [13]. Several dependent populations were compared with Friedman’s test with Dunn’s multiple comparison tests for populations of clinical importance i.e. rSO_2_-C versus rSO_2_-A on ground and in air as well as rSO_2_-C on ground versus rSO_2_-C in air and rSO_2_-A on ground and rSO_2_-A in air. The equality of scatter in two populations was performed by the Ansari–Bradley test. All statistical tests were two-sided and p values < 0.05 were considered to be statistically significant.

## Results

Electronically stored data from pediatric patients (n = 38) monitored with NIRS was investigated. The ages of transported children were: < 29 days (n = 27), 1–2 months (n = 7), > 2–3 months (n = 1), > 3–4 months (n = 1), 3–4 years (n = 2). The majority of patients (n = 21) were transported due to congenital heart disease. Eight patients were also observed in the PICU before transport, median PICU-observation time 14.1 h (IQR 9.7–16.6 h).

Demographic data, diagnosis and respiratory support are presented in Table 1.

**Table 1:** Distribution of demographic data, type of respiratory support and diagnostic groups for patients (n = 38)

Sex M/F	Age at transport Median days (IQR)	Invasive ventilation	CPAP	Spontaneous breathing	Room air/supplemental O_2_	Diagnosis: Resp/Cardiac/Misc
29/9	9.5 (4.0–33.75)	13	7	18	20/18	12/21/5

*Resp* respiratory diagnosis, *Cardiac* congenital heart disease, *Misc* miscellaneous

The median time of air-ambulance and ground-ambulance transport were 1.3 h (IQR 1.1–1.4 h) and 1.8 h (IQR 1.5–2.0 h), respectively.

**Fig. 1:**
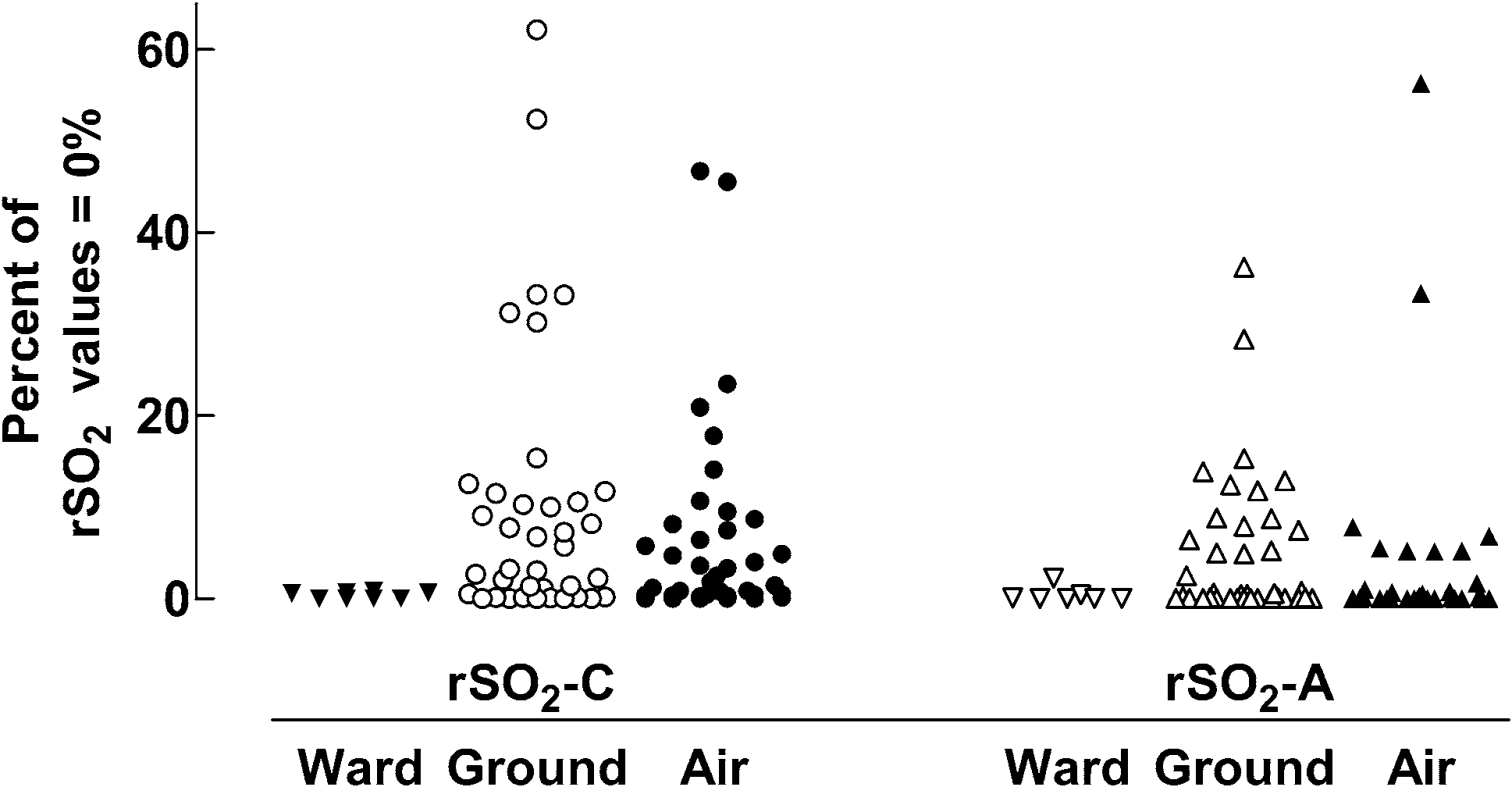
The observed percentage of rSO_2_ values = 0%, i.e. no signal, for the cerebral sensor (rSO_2_-C) and the abdominal sensor (rSO_2_-A) during pre-transport observation in the PICU, transport in ground-ambulance and in air-ambulance. Each symbol represents data from one individual patient

The observed percentage of rSO_2_ values = 0%, i.e. no signal recorded, is illustrated in Fig. 1. During pre-transport observation in the PICU, the median percentage of measurements = 0% for rSO_2_-C and rSO_2_-A, was 0.3% (IQR 0.0–0.6%) and 0.0% (IQR 0.0–0.3%) respectively. In one of the eight patients, only the cerebral sensor was applied during the pre-transport registration.

Data during transport for values = 0% analyzed with Friedman’s test showed a statistically significant difference between groups (p = 0.0002). Dunn’s multiple comparison tests revealed a statistically significant difference (p ≤ 0.01) between rSO_2_-C and rSO_2_-A during flight; i.e. a higher median percentage of zero-values in rSO_2_-C. The median percentage of measurements = 0% was 2.1% (IQR 0.4–8.2%) for rSO_2_-C during flight and 4.4% (IQR 0.2–11.6%) during ground-ambulance transport. For rSO_2_-A, the median percentage of measurements = 0% during flight was 0.0% (IQR 0.0–1.1%) and during ground ambulance transport 0.5% (IQR 0.0–8.1%) respectively.

After removal of zero-values, the occurrence of floor effects was investigated for both sensors in the PICU and during all transport phases. During pre-transport observation in the PICU there were no floor effect measurements found in the cerebral sensor. For the abdominal sensor, floor-effect values were < 1% of all values pre-transport. During transport floor effect was almost exclusively observed for rSO_2_-A. Except for one patient, floor effect values were only found in patients who had undergone abdominal surgery (n = 8, data not shown).

**Fig. 2:**
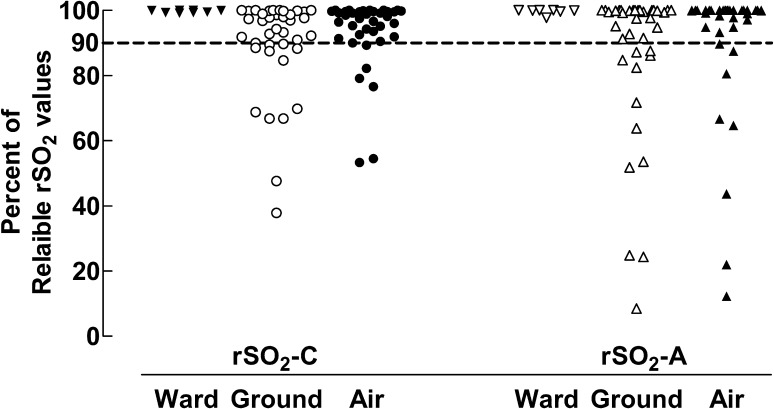
The percentage of reliable rSO_2_-C and rSO_2_-A values during pre-transport observation in the PICU, ground-ambulance transport and air-ambulance transport. The dotted line indicates 90% reliable rSO_2_ values. Each symbol represents data from one individual patient

The percentage of reliable values, which we defined as measurements without zero-values and floor effect values, was used as a measure of monitoring success and are presented in Fig. 2. Pre-transport registration in the PICU showed a high percentage of reliable values both for the cerebral and the abdominal sensors, as compared to all transport phases. The median values for reliable measurements during flight and during ground ambulance transport for rSO_2_-C were 97.8% (IQR 91.8–99.6%) and 95.6% (IQR 88.4–99.6%) respectively. For rSO_2_-A the median values were 100% (IQR 94.4–100%) and 98.5% (IQR 85.8–100%) respectively. There was no statistically significant difference between ground and flight transport for each probe.

For rSO_2_ values the degree of variation or scatter was examined. This revealed a wider scatter, expressed as the variability in percentage of reliable rSO_2_ values, for rSO_2_-A than rSO_2_-C during both ground (p = 0.022) and air transport (p = 0.005).

In addition to unreliable measurements, we noted signal noise in measurements done during transport. Therefore we decided to investigate the signal further and to reduce signal noise after all zero and floor effect values had been removed.

**Fig. 3:**
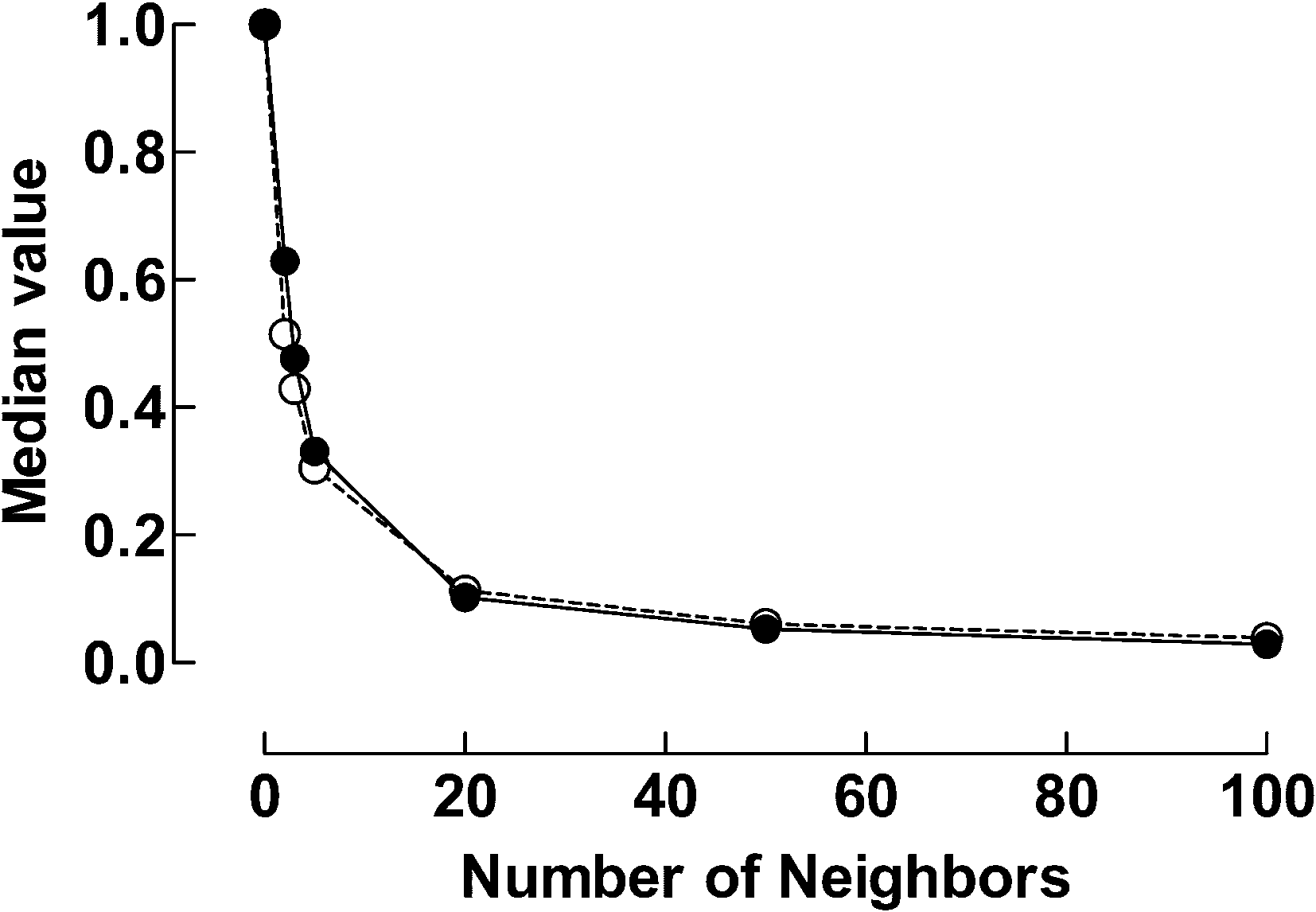
Variability in signal expressed as median value of adjacent readings as a function of number of neighbors. Line filled circle: absolute delta NIRS rSO_2_-C, dashed line open circle: absolute delta NIRS rSO_2_-A

The noise in the NIRS signal was reduced by applying the Savitzky–Golay filters. The effect of numbers of neighboring NIRS-values on the signal noise, expressed as the median absolute delta NIRS for various numbers of neighbors is illustrated in Fig. 3. The signal noise decreased markedly when the numbers of neighbors was increased up to values of 20. A further increase only seemed to affect the noise level to a minor extent. The effects of smoothing with 20, 50 and 100 neighbors respectively were tested.

**Fig. 4:**
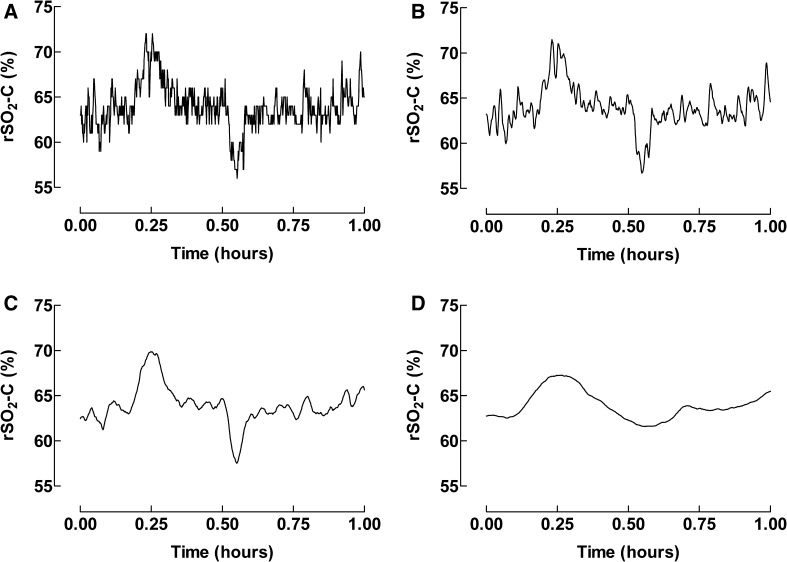
The Savitzky–Golay filtering technique used to remove noise from the signal in one patient during 1 h of transportation for rSO_2_-C. **a** Unprocessed data, **b** 5 neighbors, **c** 20 neighbors, and **d** 100 neighbors

When too many neighbors were used some peaks were smoothed away and information was lost. Too few neighbors resulted in signals with too much noise. In Fig. 4 the registration time was one hour. Significant distortions and loss of features of the data such as peaks and width were seen when the signal was smoothed with 100 neighbors.

**Fig. 5:**
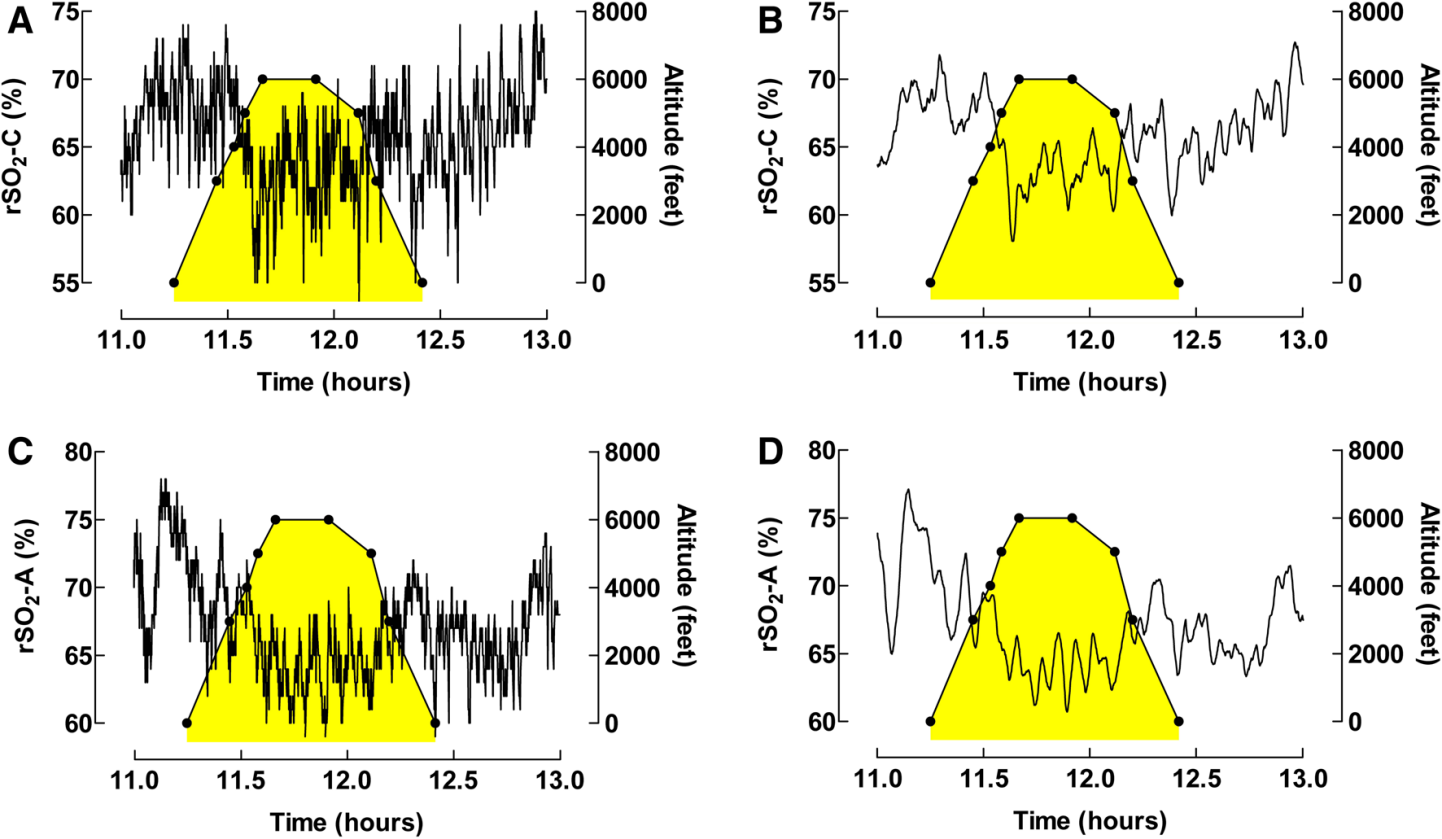
The Savitzky–Golay filtering technique used to remove noise from the signal to facilitate interpretation when rSO_2_-C and rSO_2_-A are affected by changes in altitude during flight. **a** Unprocessed data for rSO_2_-C, **b** smoothing with 20 neighbors rSO_2_-C. **c** Unprocessed data for rSO_2_-A, and **d** smoothing with 20 neighbors rSO_2_-A. The yellow-colored (shadowed) area symbolizes time in air ambulance

To facilitate signal evaluation when the rSO_2_ was affected by changes in altitude during flight, the Savitzky–Golay filtering technique was used to reduce signal noise (Fig. 5). In this patient both rSO_2_-C and rSO_2_-A measurements are shown over two hours. Smoothing with 20 neighbors was used. The NIRS measurements were seen to drop with increasing altitude, both in the cerebral and the abdominal sensors.

## Discussion

To our knowledge, this is the first time a thorough clinical evaluation of the NIRS-signal during pediatric transport is performed.

We found that NIRS measurements proved feasible both during ground and air transport for both cerebral and abdominal measurement sites. The proportion of reliable values was lower during transport than for measurements done in the hospital setting.

Disturbances in the NIRS-signal were different for the two sensors. Zero measurement (NIRS = 0%) occurred more often in rSO_2_-C measurements during air transport. Possible reasons could be bright ambient light, poor probe adhesion or placement over hair (Fig. 1).

Floor effect was almost exclusively observed for rSO_2_-A during transport. The presence of long periods of rSO_2_ measurements of 15%, “floor effects”, without clinical deterioration has been described by others who also recommended caution in interpretation of these low values [14]. The majority of patients in whom floor effect occurred had undergone abdominal surgery. The cause of this observation might be scarring of the subcutaneous tissue and possible inferior penetrating conditions for the near-infrared light into the underlying tissue but this relationship should be examined in a larger group of patients. Clinicians should be aware of these situations in order to make decisions based on the clinical context as well as monitor values.

To decrease signal noise and to enable better signal evaluation; i.e. to increase the specificity in the signal, we decided to examine if post-hoc processing could be used. After removal of all 0 and 15% values, we used the Savitzky–Golay algorithm for smoothing of the electronically stored data to perform noise reduction in the signal with the lowest possible signal distortion. This smoothing algorithm is a feasible technique to improve the signal-to-noise ratio in any kind of signal [15]. The method has been advocated to preserve features of the data, such as widths and heights of peaks, better than average filtering methods. It has been used in different disciplines such as analytical chemistry, forensic science and satellite data analysis [15].

In this study, we have shown that the Savitzky–Golay algorithm can also be used to facilitate interpretation of changes in the NIRS signal in relation to physiological changes in patients and events during transport. When the noise is reduced, it is less likely that changes are related to disturbances in signal acquisition than to physiological events. By using the algorithm with Matlab software, the data points are not required to have uniform spacing. We found that the algorithm could reduce signal-to-noise ratio without distorting the underlying information at a window-size of 20–50 neighbors (Fig. 3).

The effect of hypoxia at altitude is a commonly discussed area of risk regarding medical transports. NIRS may provide information regarding oxygenation from tissues which are potentially affected during transport in air ambulance. Taking advantage of the methods presented in this study it is possible to evaluate physiological changes as well as adverse events during transport based on NIRS data (Fig. 5). Possible applications include quality assurance work, study of the course of adverse events and the possibility to connect for example ventilator settings with the NIRS signal in Patient Data Management Systems (PDMS). Most importantly, this method provides a unique opportunity to evaluate in detail physiological processes during transport.

While reducing noise is the goal of filtering, there is a risk that some of the ‘noise’ may actually represent true events, e.g., a short-term drop in tissue saturation which quickly responds to therapy. In other words, by applying a filter there is a risk that increasing specificity occurs at a cost to sensitivity, as exemplified in Fig. 4. Caution must be used in determining an appropriate degree of data processing. Additionally, only relying on processed data for post-hoc analyses of the transport might obscure true events, and it is therefore important to relate the post-hoc analyses to clinical data and documentation. Other limitations in this study include comparison of in-hospital to transport data. Measurements in hospital pre-transport seemed to be more reliable than measurements during transport, but the number of patients observed in hospital was small. This was partly due to the nature of the patients’ conditions and their need for acute inter-hospital transports.

## Conclusion

NIRS measurements proved feasible during inter-hospital transfer of critically ill pediatric patients in ground and air ambulance transports for both cerebral (rSO_2_-C) and abdominal (rSO_2_-A) measurement sites. The occurrence of unreliable measurements increased during transport. Filtering of the signal by use of the Savitzky–Golay algorithm improved the signal-to-noise ratio in the near-infrared signal without distorting the underlying information at a window-size of 20–50 neighbors. This study shows that the electronically stored data can be filtered and assessed off-line after the transport and provide valuable post-hoc information about the transport, information which can be used in research as well as quality assurance evaluation of patient transports.

## References

[1] Jobsis FF (1977). Noninvasive, infrared monitoring of cerebral and myocardial oxygen sufficiency and circulatory parameters. Science.

[2] Murkin JM, Arango M (2009). Near-infrared spectroscopy as an index of brain and tissue oxygenation. Br J Anaesth 103 Suppl.

[3] Scott JP, Hoffman GM (2014). Near-infrared spectroscopy: exposing the dark (venous) side of the circulation. Paediatr Anaesth.

[4] Nagdyman N, Fleck T, Schubert S, Ewert P, Peters B, Lange PE, Abdul-Khaliq H (2005). Comparison between cerebral tissue oxygenation index measured by near-infrared spectroscopy and venous bulb saturation in children. Intensive Care Med.

[5] Kaufman J, Aldomovar MC, Zuk J, Friesen RH (2008). Correlation of abdominal site near-infrared spectroscopy with gastric tonometry in infants following surgery for congenital heart disease. Pediatr Crit Care Med.

[6] Booth EA, Dukatz C, Ausman J, Wider M (2010). Cerebral and somatic venous oximetry in adults and infants. Surg Neurol Int.

[7] Genbrugge C, Dens J, Meex I, Boer W, Eertmans W, Sabbe M, Jans F, De Deyne C (2016). Regional cerebral oximetry during cardiopulmonary resuscitation: useful or useless?. J Emerg Med.

[8] Stroud MH, Gupta P, Prodhan P (2012). Effect of altitude on cerebral oxygenation during pediatric interfacility transport. Pediatr Emerg Care.

[9] Valente ME, Sherif JA, Azen CG, Pham PK, Lowe CG (2016). Cerebral oxygenation and acceleration in pediatric and neonatal interfacility transport. Air Med J.

[10] Burillo-Putze G, Herranz I, Pérez V, Redondo F, Fernández F, Jiménez-Sosa A, Alvarez J (2002). Transcranial oximetry as a new monitoring method for HEMS. Air Med J.

[11] Weatherall A, Skowno J, Lansdown A, Lupton T, Garner A (2012). Feasibility of cerebral near-infrared spectroscopy monitoring in the pre-hospital environment. Acta Anaesthesiol Scand.

[12] Hamrin TH, Berner J, Eksborg S, Radell PJ, Fläring U (2016). Characteristics and outcomes of critically ill children following emergency transport by a specialist paediatric transport team. Acta Paediatr.

[13] Savitzky A, Golay MJE (1964). Smoothing and differentiation of data by simplified least squares procedures. Anal Chem.

[14] McNeill S, Gatenby JC, McElroy S, Engelhardt B (2011). Normal cerebral, renal and abdominal regional oxygen saturations using near-infrared spectroscopy in preterm infants. J Perinatol.

[15] Vivó-Truyols G, Schoenmakers PJ (2006). Automatic selection of optimal Savitzky–Golay smoothing. Anal Chem.

